# 2023 Electrophysiology Literature in Review: A Surgeon’s Perspective

**DOI:** 10.19102/icrm.2024.15015

**Published:** 2024-01-15

**Authors:** Sarah Yousef, Andy C. Kiser

**Affiliations:** 1 Department of Cardiothoracic Surgery, University of Pittsburgh School of Medicine, Pittsburgh, PA, USA; 2 Cardiovascular Services, St. Clair Health, Pittsburgh, PA, USA

In 2023, we have had several noteworthy publications concerning surgery and rhythm management. Two studies—a retrospective institutional database analysis^1^ and a literature review with meta-analysis^2^—examined the outcomes for transcatheter aortic valve replacement (TAVR) compared to redo surgical aortic valve replacement (SAVR) for failed surgical aortic bioprostheses. The authors included a comparison of permanent pacemaker (PPM) implantation rates between the two groups. The 5-year results of the aMAZE trial^3^ followed up on the previously reported 2-year results of concomitant atrial fibrillation (AF) surgery during cardiac operations with respect to the maintenance of sinus rhythm (SR), survival, and quality of life (QOL). In the Posterior Left Pericardiotomy for the Prevention of Atrial Fibrillation After Cardiac Surgery (PALACS) trial, the authors performed a post hoc analysis of their study population.^4^ They reported characteristics, variables, and outcomes of postoperative AF (POAF) and evaluated the impact of a posterior pericardiotomy (PP) on POAF. Finally, the Cardiothoracic Surgical Trials Network examined pacemaker implantation rates for patients randomized to either mitral valve surgery (MVS) or to MVS with tricuspid annuloplasty (TA).^5^ This summary reviews these important publications from 2023.

## Permanent pacemaker rates following valve-in-valve transcatheter aortic valve replacement may be similar to or lower than those following redo surgical aortic valve replacement

The retrospective institutional database analysis by Yousef et al.^1^ compared 198 patients who underwent valve-in-valve (ViV) TAVR with 147 patients who underwent redo SAVR. Five patients in the ViV TAVR group required a PPM (2.5%), whereas 15 patients in the redo SAVR group required a PPM (10.2%; *P* = .002).

A systematic review and meta-analysis by Raschpichler et al.^2^ compared outcomes of ViV TAVR to redo SAVR across 15 studies with a total of 8881 patients, half (50.2%) of whom underwent ViV TAVR and half of whom underwent redo SAVR. In this meta-analysis, PPM rates did not differ significantly between patients undergoing ViV TAVR and those undergoing redo SAVR (risk ratio, 0.76; *P* = .21).

## Concomitant atrial fibrillation surgery confers improved sinus rhythm without a significant impact on stroke rates or quality of life at 5 years

The aMAZE multicenter, randomized, controlled trial^3^ performed a prospective randomization of 352 non-emergent patients to cardiac surgery with concomitant AF surgery or cardiac surgery alone. The 2-year results, while demonstrating an increased probability of SR for patients with concomitant AF surgery, failed to show an improvement in the incidence of stroke, survival, or QOL despite the recovery of atrial contractile function in those who returned to SR. To further elucidate the long-term advantages of adding AF surgery, the group examined the 5-year results of their study population. All patients had AF for >3 months prior to surgery and were randomized on the day of the procedure. All cardiac surgical procedures were included, and the surgeon determined the ablation lesion set. Because 93.2% of patients at 2 years on 4-day continuous electrocardiogram (ECG) monitors were either wholly in AF or wholly in SR, only a 12-lead ECG at 5 years was used to determine rhythm. QOL was measured using the EuroQol EQ-5D-3 questionnaire administered serially from randomization to 5 years.

At the time of the report, 182 patients had completed the 5-year follow-up (AF surgery, 92 patients; control, 90 patients). Of the 132 patients with ECG data available at 5 years, 38.9% of patients in the concomitant AF group and 22.4% of patients in the surgery-alone group were in SR (*P* = .044), with a 2.21 odds ratio for SR at 5 years. Fewer patients in the concomitant AF group had strokes (12 vs. 19 patients), but this difference did not reach statistical significance (*P* = .22). Almost 50% of strokes (7/12 AF surgery; 9/19 control) occurred between years 2–5. When the outcomes were adjusted for the length of follow-up, the stroke rate per year was 1.6% for the AF surgery group and 2.5% for the control group, respectively, with an odds ratio of 0.625. The Kaplan–Meier curve for mortality demonstrated increased mortality rates in the first 3 months for both groups with a non-significant separation of survival rates between years 1–4.5 post-procedure that favored the control group. However, the rate of survival at 5 years was essentially the same in both groups (AF surgery, 77.3%; control, 77.8%). Neither age nor procedure impacted survival between groups. Ninety-three percent of patients contributed at least two measurements in the QOL survey for both groups. There was no difference in QOL detected at any point during the 5-year follow-up between the groups.

The authors noted that half of the patients were lost to follow-up at 5 years, but they reported a similarly proportional loss in each group, thereby limiting bias in the comparison. They also recognized the poorly controlled lesion set choice by the surgeons and an inability to classify the type of AF in the study population (paroxysmal, persistent, or long-standing persistent). However, the findings of this “real-world” analysis demonstrate that, despite a higher rate of SR with the addition of AF surgery to cardiac surgical procedures, there was no important difference in stroke, mortality, or QOL at 5 years.

## Posterior pericardiotomy during cardiac surgical procedures reduces postoperative atrial fibrillation

The PALACS trial provided a post hoc analysis of a single-center, randomized trial comparing patients who received a PP to those without a PP during cardiac surgery.^4^ The trial described the clinical and hemodynamic characteristics of POAF. Patients with mitral or tricuspid disease were excluded, and 89.3% of patients received β-blocker therapy beginning day 1 after surgery.

Of the 420 patients enrolled, 103 (24.5%) developed POAF, with the majority doing so on the second and third postoperative days (PODs) (40.8% and 30.1%, respectively). The median duration of POAF was 24 h, with 70.9% of patients returning to SR within 36 h. Most patients (97.1%) received anti-arrhythmic therapy, with only 23 patients (22.3%) requiring electrical cardioversion, including nine patients for hemodynamically unstable tachycardia. All patients returned to SR at 30 days of follow-up. A multivariable logistic regression analysis demonstrated that older age was significantly associated with POAF, while postoperative β-blockers, female sex, and PP predicted decreased rates of POAF. Patients with PPs had a lower rate of POAF compared to those without PPs (17.7% vs. 31.3%, *P* = .001).

The authors concluded that, unlike in previous studies, continuous ECG monitoring during postoperative hospitalization improved the detection of POAF in their study. The reduced incidence of POAF in the PP group, especially at POD 2, suggests that the later episodes of AF may be related to pericardial effusions, while early episodes may be due to intrinsic arrhythmogenic substrates. Although patients who developed POAF experienced a prolonged hospital stay, there was no difference in mortality between the two groups at 30 days. The study is limited to 30-day outcomes; longer-term evaluations of the incidence of recurrent AF, stroke, and mortality are ongoing.

## Tricuspid annuloplasty during mitral valve surgery portends an increased risk of pacemaker implantation

The Cardiothoracic Surgical Trials Network performed a randomized trial of patients with moderate tricuspid regurgitation (TR) and patients with tricuspid annular dilation and mild TR who were undergoing surgical mitral valve repair or replacement to either MVS alone or MVS with TA.^5^ The index analysis demonstrated a lower 2-year rate of TR progression but failed to demonstrate a clinical effect on mortality, symptoms, or rehospitalization. However, the investigators reported a significantly higher rate of PPM implantation in the TA patients. This current report re-evaluated the original trial data to assess the risk factors for PPM in the study population.

The initial study enrolled 401 patients, including 198 patients who underwent MVS with TA and 203 who underwent MVS alone. Thirty-six patients (9.6%) received a PPM within the 2-year follow-up. Complete or high-grade atrioventricular block led to PPM implantation in most patients (52.8%), with 30.6% requiring a PPM due to sinus node dysfunction. In the MVS with TA group, 30 (16%) patients had a PPM, while only six patients (3.2%) needed a PPM in the MVS-alone group (*P* < .001). Most PPM implantations (30/36; 80.6%) occurred within 30 days of the procedure. Age and TA were independently associated with a PPM requirement within 30 days, while age, TA, and ejection fraction were independently associated with PPM implantation at 2 years.

While patients who underwent MVS with TA experienced a lower incidence of moderate-to-severe TR compared to those with MVS alone (3.4% vs. 25.1%), the benefit carried a significantly higher PPM requirement. However, those patients with moderate or less TR who received TA had lower composite rates of death, reoperation for TR, or progression of TR compared to those with MVS alone (3.9% vs. 10.2%; *P* = .02). The authors could not demonstrate a correlation of this increased PPM requirement to TA ring size or to the type of ring selected (semi-rigid or flexible). Although surgical technique, surgeon experience, and associated Maze procedures were not components of the PPM evaluation, the paper’s discussants parenthetically emphasized these to be the most important contributing factors leading to PPM implantation.

## Authors’ comments

A systematic review of pacemaker rates after TAVR by Van Rosendael et al.^6^ for patients undergoing initial TAVR, not ViV, reports PPM implantation rates of 2.3%–36.1%. The newest-generation SAPIEN 3 (Edwards Lifesciences, Irvine, CA, USA) balloon expandable devices carry a PPM incidence of 4%–24%, while the newest-generation self-expanding Evolut-R (Medtronic, Minneapolis, MN, USA) devices experience a PPM incidence of 14.7%–26.7%. While both studies reviewed here examined many valve-related outcome variables, both agree that ViV TAVR patients experience lower PPM rates than index, non-ViV, TAVR patients and have at least similar—if not lower—PPM rates than redo SAVR patients.

The aMAZE investigators followed up on their 2-year results by releasing 5-year data in an attempt to explain the higher-than-expected incidence of stroke and decreased QOL despite a return to SR. Unfortunately, this analysis failed to show a positive impact of “real-world” concomitant AF procedures on death, stroke, or QOL. Variability in ablation patterns and the type of AF may have played a role in these results, but adding concomitant AF surgery did improve the rates of SR at 5 years and remains a class IIa, “A” Level of Evidence guideline.^7^

The PALACS trial provides detailed clinical characteristics of POAF, highlighting the importance of continuous monitoring post-procedure until discharge. The authors’ improved rate of POAF in patients with PP supports their suggestion that delayed AF events may be related to postoperative inflammatory mediators within retained pericardial fluid.

As surgeons consider TA for patients undergoing MVS, one must consider the sixfold increased PPM risk with respect to the long-term benefits of treating TR. The addition of a Maze procedure for AF or TA for significant tricuspid pathology should not be abandoned given the findings of the reports reviewed here. Instead, we should pursue these concomitant interventions when clinically warranted and with sound judgment. By advancing our expertise, following our results, and reviewing our outcomes with our peers, we will provide accountable care to our patients.
